# Comparison of humoral and cellular immune responses in hematologic diseases following completed vaccination protocol with BBIBP-CorV, or AZD1222, or BNT162b2 vaccines against SARS-CoV-2

**DOI:** 10.3389/fmed.2023.1176168

**Published:** 2023-07-17

**Authors:** Enikő Szabó, Szabolcs Modok, Benedek Rónaszéki, Anna Faragó, Nikolett Gémes, Lajos I. Nagy, László Hackler, Katalin Farkas, Patrícia Neuperger, József Á. Balog, Attila Balog, László G. Puskás, Gabor J. Szebeni

**Affiliations:** ^1^Laboratory of Functional Genomics, Biological Research Centre, Szeged, Hungary; ^2^Department of Medicine, Szent-Györgyi Albert Medical School-University of Szeged, Szeged, Hungary; ^3^Avidin Ltd., Szeged, Hungary; ^4^Doctoral School in Biology, Faculty of Science and Informatics, University of Szeged, Szeged, Hungary; ^5^AstridBio Technologies Ltd., Szeged, Hungary; ^6^Department of Rheumatology and Immunology, Faculty of Medicine, Albert Szent-Gyorgyi Health Centre, University of Szeged, Szeged, Hungary; ^7^Avicor Ltd., Szeged, Hungary; ^8^Department of Physiology, Anatomy and Neuroscience, Faculty of Science and Informatics, University of Szeged, Szeged, Hungary; ^9^CS-Smartlab Devices, Kozarmisleny, Hungary

**Keywords:** hematology diseases, SARS-CoV-2 vaccination, BBIBP-CorV, AZD1222, BNT162b2, COVID-19, protective immunity

## Abstract

**Background:**

Vaccination has proven the potential to control the COVID-19 pandemic worldwide. Although recent evidence suggests a poor humoral response against SARS-CoV-2 in vaccinated hematological disease (HD) patients, data on vaccination in these patients is limited with the comparison of mRNA-based, vector-based or inactivated virus-based vaccines.

**Methods:**

Forty-nine HD patients and 46 healthy controls (HCs) were enrolled who received two-doses complete vaccination with BNT162b2, or AZD1222, or BBIBP-CorV, respectively. The antibodies reactive to the receptor binding domain of spike protein of SARS-CoV-2 were assayed by Siemens ADVIA Centaur assay. The reactive cellular immunity was assayed by flow cytometry. The PBMCs were reactivated with SARS-CoV-2 antigens and the production of activation-induced markers (TNF-α, IFN-γ, CD40L) was measured in CD4^+^ or CD8^+^ T-cells *ex vivo*.

**Results:**

The anti-RBD IgG level was the highest upon BNT162b2 vaccination in HDs (1264 BAU/mL) vs. HCs (1325 BAU/mL) among the studied groups. The BBIBP-CorV vaccination in HDs (339.8 BAU/mL ^***^p < 0.001) and AZD1222 in HDs (669.9 BAU/mL *p < 0.05) resulted in weaker antibody response vs. BNT162b2 in HCs. The response rate of IgG production of HC vs. HD patients above the diagnostic cut-off value was 100% vs. 72% for the mRNA-based BNT162b2 vaccine; 93% vs. 56% for the vector-based AZD1222, or 69% vs. 33% for the inactivated vaccine BBIBP-CorV, respectively. Cases that underwent the anti-CD20 therapy resulted in significantly weaker (^**^p < 0.01) anti-RBD IgG level (302 BAU/mL) than without CD20 blocking in the HD group (928 BAU/mL). The response rates of CD4^+^ TNF-α^+^, CD4^+^ IFN-γ^+^, or CD4^+^ CD40L^+^ cases were lower in HDs vs. HCs in all vaccine groups. However, the BBIBP-CorV vaccine resulted the highest CD4^+^ TNF-α and CD4^+^ IFN-γ^+^ T-cell mediated immunity in the HD group.

**Conclusion:**

We have demonstrated a significant weaker overall response to vaccines in the immunologically impaired HD population vs. HCs regardless of vaccine type. Although, the humoral immune activity against SARS-CoV-2 can be highly evoked by mRNA-based BNT162b2 vaccination compared to vector-based AZD1222 vaccine, or inactivated virus vaccine BBIBP-CorV, whereas the CD4^+^ T-cell mediated cellular activity was highest in HDs vaccinated with BBIBP-CorV.

## 1. Introduction

After the COVID-19 outbreak, it has rapidly become clear that SARS-CoV-2 infection is a higher threat with more severe clinical course to patients with hematological diseases (HD). Patients with HD suffer from higher mortality rate than the general population with COVID-19 or non-hematology COVID-19 patients (1–3) which can be explained by risk factors such as age, comorbidities and immunosuppressive therapies. After SARS-CoV-2 infection nearly one-third of the patients (31%) has been reported to be serologically negative for SARS-CoV-2 IgGs (4).

The vaccination against severe acute respiratory syndrome coronavirus 2 (SARS-CoV-2) emerged as the first line defense strategy in the fight against the global pandemic, and available vaccines have prevented COVID-19 related hospitalization, severe disease and death worldwide (5). SARS-CoV-2 vaccination improved the mortality rate of HD patients from 31% (pre-vaccination era) to 9% (6) which is still remarkably higher compared to the rate observed in the fully vaccinated overall population (7). A recent meta-analysis including 26 studies revealed that patients with HD had significantly lower seroconversion rate (33.3%) compared to healthy controls (74.9%) and despite the increase of seroconversion rates after the second dose, the significant difference between the two groups remained (65.3% vs. 97.8%) (8). A more recent meta-analysis of 150 studies with 20922 HD patients showed a pooled seroconversion rate after SARS-CoV-2 vaccination of 67.7% and the meta-regression analysis revealed that patients with lymphoid malignancies, but not myeloid malignancies, had lower seropositivity rates than those with solid tumors (9).

Multiple studies highlighted that patients with hematological malignancies receiving immunosuppressive therapies such as stem cell transplantation, anti-CD20 therapies, Bruton’s tyrosine kinase (BTK) inhibitors and CAR-T cell treatments are at higher risk. Anti-CD20 monoclonal antibody therapy may result in prolonged depletion of normal B-cells and therefore markedly impaired humoral response to COVID-19 vaccination in HD patients with undetectable or decreased protective antibody titers (10–14).

There are several aspects of vaccination efficacy regarding this vulnerable group of patients which are rarely discussed in the literature. First, beyond the scope of humoral response to COVID-19 vaccination, the role of vaccine-induced cellular response is less explored. Minority of published articles incorporated the assessment of SARS-CoV-2-specific T-cell response after complete vaccination with detectable T-cell mediated immunity ranging between 29% and 88% of the HD patients (9, 10, 12, 15–17). Second, majority of the reports demonstrated the efficacy of mRNA and vector-based vaccines, although in many countries (including Hungary) inactivated vaccines are also approved.

To narrow down this knowledge gap, we conducted this complex study to compare the immunogenicity of mRNA (BNT162b2), vector-based (AZD1222) and inactivated (BBIBP-CorV) vaccine in a cohort of fully vaccinated patients with HD versus healthy individuals. Both humoral and cellular immunity was evaluated by measuring the neutralizing anti-SARS-CoV-2 antibody titers and by quantifying SARS-CoV-2 reactive T-cells with the help of multicolor flow cytometry.

## 2. Materials and methods

### 2.1. Ethical statement

The enrollment of patients was reviewed and approved by the Human Investigation Review Board of the National Public Health Center under Project Identification Code 47226-7/2019EÜIG. The patients provided their written informed consent to participate in this study. Subjects were informed about the study by a physician and acute SARS-CoV-2 infection was ruled out by RT-qPCR. Laboratory studies and interpretations were performed on coded samples lacking personal and diagnostic identifiers. The study adhered to the tenets of the most recent revision of the Declaration of Helsinki.

### 2.2. Study population

The main characteristics of the study participants (46 healthy controls, HCs and 49 hematologic disease patients, HDs) are summarized Table 1 and detailed demographic data of the enrolled HD patients vaccinated with BBIBP-CorV are summarized in Supplementary Table S1, vaccinated with AZD1222 are summarized in Supplementary Table S2, vaccinated with BNT162b2 are summarized in Supplementary Table S3. All participants received two doses of the relevant vaccine in line with recommendations of the respective manufacturer of BBIBP-CorV (Sinopharm, Beijing Institute, Beijing, China); AZD1222 (ChAdOx1, University of Oxford and AstraZeneca, Cambridge, United Kingdom); and BNT162b2 (Comirnaty, Pfizer-BioNtech, Mainz, Germany). This prospective observational study was conducted at the Szent-Györgyi Albert Medical School-University of Szeged, Department of Medicine, Szeged, Hungary between October 2021 and February 2021. Adult patients with HDs were recruited who received two doses vaccination starting from February 2021 and completed by 1st June 2021. Peripheral blood and sera sampling was conducted after 4 months of the second vaccination event. The schematic cartoon of the project workflow is demonstrated in Supplementary Figure S1.

**Table 1:** Demographic data of the enrolled HCs and HD patients vaccinated with anti-SARS-CoV-2 specific vaccines.

	Age (years, mean ± SD)	Female	BBIBP-CorV	AZD1222	BNT162b2	Therapy: anti-CD20	Disease duration (years, mean ± SD)
All HCs, *n* = 46	43 ± 12	*n* = 30 (65%)	*n* = 16 (35%)	*n* = 14 (30%)	*n* = 16 (35%)	–	–
All patients with HDs, *n* = 49	63 ± 14	*n* = 20 (40%)	*n* = 15 (30%)	*n* = 16 (33%)	*n* = 18 (37%)	*n* = 11 (22%)	4.2 ± 3.2

This study is a cross sectional analysis including a wide variety of hematology patients. Patients diagnosed with acute leukemia and aggressive lymphoma under induction chemotherapy were excluded. Treatment with tyrosine kinase inhibitors such as ibrutinib, ruxolitinib and dasatinib was allowed, similarly to any kind of anti-myeloma treatment including intermittent corticosteroids. A subgroup with known prior anti-CD20 moAb treatment was created. Patients with any sign of an acute infection, including confirmed acute SARS-CoV-2 infection were excluded. The distribution of vaccine types represents vaccine usage in the entire hematology patient population.

The withdrawal of 10 mL peripheral blood was carried-out into Lithium Heparin tubes (BD vacutainer, Beckton Dickinson). The primary endpoint was the humoral and cellular immunogenicity of homologous two doses of BBIBP-CorV or AZD1222, or BNT162b2, respectively. Secondary endpoints included: effect of anti-CD20 treatment and hematologic disease duration on the production of anti-RBD neutralizing antibodies in HD patients.

### 2.3. Measurement of anti-SARS-CoV-2 IgG antibodies

Measurement of SARS-CoV-2 anti-RBD (receptor binding domain) of spike (S) protein, the IgG-type antibodies was performed as described in detail previously by our group (18, 19). Briefly, quantitative measurement of neutralizing anti-RDB specific IgG-type antibody levels was performed with the Siemens Advia Centaur XPT system using the Siemens Healthineers SARS-CoV-2 IgG assay (sCOVG) (Siemens Healthineers, Munich, Germany). Irsara et al. (20), showed a proper correlation (*r* = 0.84) of the positive sCOVG assay results with virus neutralization capacity. Measured index values were converted into WHO 20/136 approved international units of 1000 Binding Antibody Unit per milliliter (BAU/mL) using the following equation: (sCOVG index) × 21.8 = BAU/mL, where the diagnostic cut-off value was 21.8 BAU/mL), assay sensitivity was 10.9 BAU/mL (21).

### 2.4. Measurement of SARS-CoV-2 specific cellular immunity

Measurement of SARS-CoV-2 specific T-cell mediated immunity was performed as described in detail previously by our group (18, 19). Briefly, measurement of SARS-CoV-2 specific T-Cell memory was performed according to the instruction of the manufacturer using the SARS-CoV-2 Prot_S T Cell Analysis Kit (PBMC) (Miltenyi Biotec, Cat. No.: 130-127-586). The PBMCs were isolated by gradient centrifugation using Leucosep tubes (Greiner Bio-One, Cat. No.: 163288) following the instructions of the manufacturer. After the isolation of PBMCs and *in vitro* stimulation, staining with the antibodies, a minimum of 2 × 10^5^ CD3^+^ cells were acquired on CytoFLEX S FACS (Beckman Coulter). Manual gating was used to determine CD4^+^ or CD8^+^ T-cells within live CD14^−^/CD20^−^, CD3^+^ lymphocytes in CytExpert (Beckman Coulter). Reactive cells were gated as CD4^+^ TNF-α^+^, CD4^+^ IFN-γ^+^, CD4^+^ CD40L^+^, CD8^+^ TNF-α^+^ and CD8^+^ IFNγ^+^ upon stimuli with the following pool of SARS-CoV-2 derived synthetic peptides: S-(spike, PepTivator SARS-CoV-2 Prot_S, Cat. No.: 130-126-701), M-(membrane, PepTivator SARS-CoV-2 Prot_M, Cat. No.: 130-126-702), N-(nucleocapsid, PepTivator SARS-CoV-2 Prot_N, Cat. No.:130-126-698) according to the instructions of the manufacturer (Miltenyi Biotec). The controls were the patient matched PBMCs left untreated. Gating was above the negative cells in the untreated control samples analyzed individually for each patient. The gating strategy has already been published by our group in the Supplementary Figure S1 in the reference Szebeni et al. (18). Cell numbers in the reporting gates were normalized to parental CD4^+^ or CD8^+^ T-cells (reactive cell number/parental cell number × 10^6^), then the background was normalized via subtraction of untreated from the stimulated. Finally, reactive cell numbers are shown in relation to 10^6^ CD4^+^ or CD8^+^ T-cells (Mean ± SEM/1 × 10^6^ parental CD4^+^ T-cells, SD), the cut-off value was 400 reactive cells of 10^6^ parental population.

### 2.5. Statistics

Data were analyzed with GraphPad Prism 8.0.1. Normality of distributions were tested with D’Agostino & Pearson test with an 0.05 alpha value. None of the groups were normally distributed datasets, so we used non-parametric Mann–Whitney test for two group comparisons and Kruskal–Wallis test was applied for three group comparisons. Dunn’s test was used for multiple comparisons. Differences are considered significant at ^*^p < 0.05, ^**^p < 0.01, and ^***^p < 0.001.

## 3. Results

### 3.1. SARS-CoV-2 specific humoral immunity in HDs following vaccination

**Figure 1:**
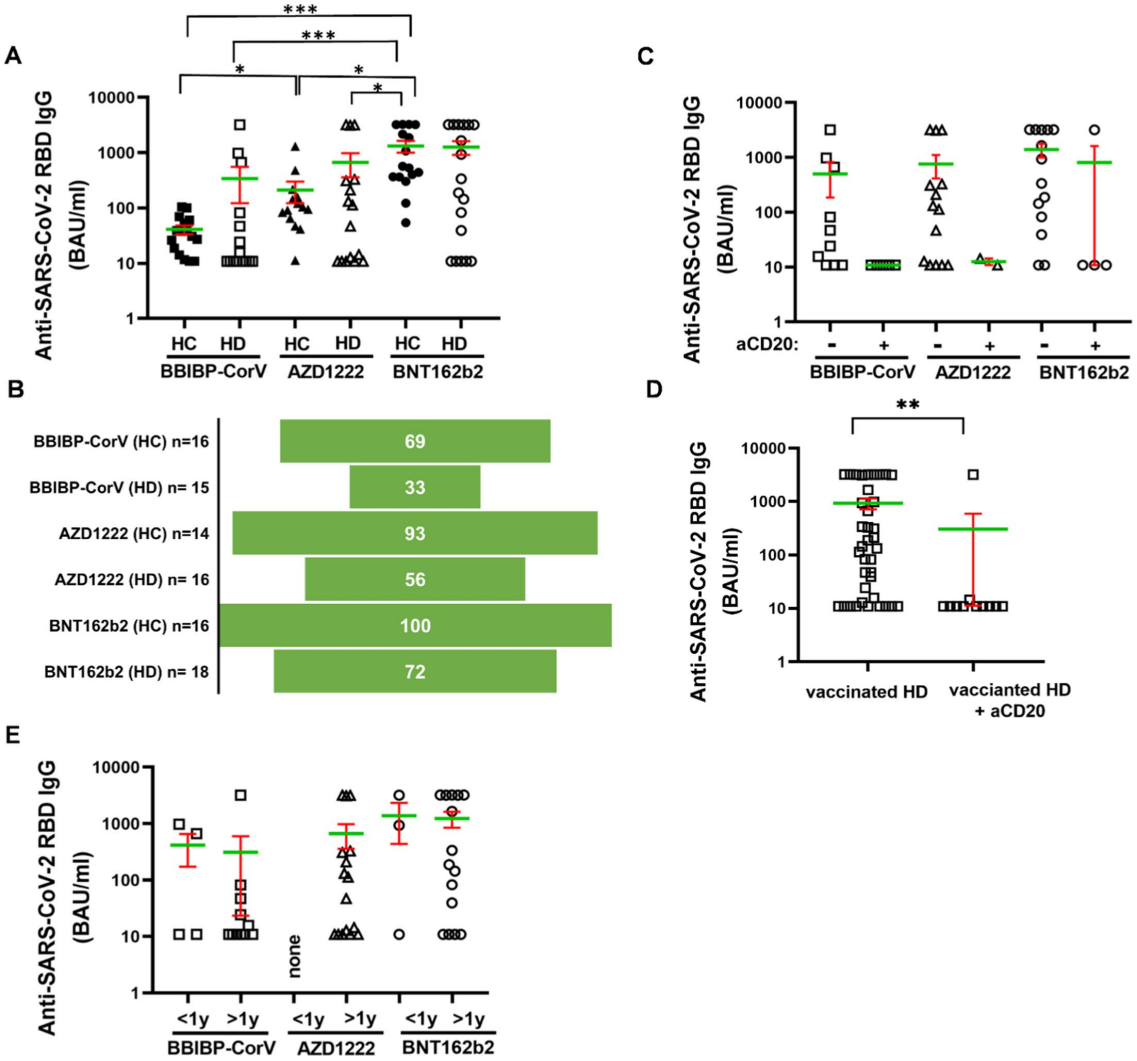
Humoral immune response to SARS-CoV-2 in HDs vs. HCs. The IgG-type anti-RBD (spike) antibodies were measured from the sera of the patients. **(A)** Subjects for studying BBIBP-CorV were *n* = 16 (HC) and *n* = 15 (HD), for AZD1222 were *n* = 14 (HC) and *n* = 16 (HD) for BNT162b2 were *n* = 16 (HC) and *n* = 18 (HD). **(B)** The response rate was calculated of the vaccination groups. Sub-group analysis was carried-out focusing on **(C,D)** anti-CD20 treatment or **(E)** disease duration. Mean (green line) and SEM (red whisker) values are demonstrated. ^*^p < 0.05, ^**^p < 0.01, and ^***^p < 0.001.

The receptor binding domain (RBD) specific anti-spike (S) IgG isotype antibodies were measured in HDs versus healthy controls following complete vaccination after 4 months. Three types of SARS-CoV-2 specific vaccines were tested, the inactivated vaccine (BBIBP-CorV), or adenovirus vector-based (AZD1222), or mRNA (BNT162b2) technology-based vaccines (Figure 1A). Dunn’s test was used for multiple comparisons and significant differences are marked in Figure 1. The humoral response rate corresponds to the percentage of subjects in one group in terms of the production of SARS-CoV-2 reactive IgG antibodies over the cut-off value 21.8 BAU/mL. The simple ‘response’ in Results 3.1 corresponds to SARS-CoV-2 reactive IgG production. The anti-RBD IgG level (mean of the BAU/mL ± SEM, SD) was the highest upon BNT162b2 vaccination in HDs (1264 ± 348.6, SD: 1479) vs. HCs (1325 ± 318.9, SD: 1276) among the studied groups. The BBIBP-CorV vaccination in HDs (339.8 ± 216.9, SD: 840 ^***^p < 0.001) and AZD1222 in HDs (669.9 ± 310.5, SD: 1242) ^*^p < 0.05) resulted in weaker antibody response vs. BNT162b2 in HCs (1325 ± 318.9, SD: 1276). The BBIBP-CorV in HCs showed less anti-RBD antibody production (41.09 ± 7.4, SD: 30 ^*^p < 0.05) vs. AZD1222 in HCs (211.8 ± 89.48, SD: 335), or vs. BNT162b2 in HCs (1325 ± 318.9, SD: 1242 ^***^p < 0.001) respectively (Figure 1A). The humoral response rate of HC vs. HD patients above the diagnostic cut-off value was 69% vs. 33% for the inactivated vaccine BBIBP-CorV; 93% vs. 56% for the vector-based AZD1222, or 100% vs. 72% for the mRNA-based BNT162b2 vaccine (Figure 1B), respectively.

Anti-CD20 therapy was given in the 22% (*n* = 11) of HDs (Table 1). The relatively low number of subjects (BBIBP-CorV:5; AZD1222:2, BNT162b2:4) receiving anti-CD20 therapy did not allow statistical comparisons of anti-RBD antibody levels between the vaccination groups. Although only one of eleven HD patients crossed the cut off 21.8 BAU/mL value in the anti-CD20 therapy group irrespectively of the type of vaccines. On the contrary, 27 of 38 HDs (71%) showed positive response rate to vaccination without CD20 blocking therapeutics (Figure 1C). Pooling data of cases underwent the anti-CD20 therapy resulted in significantly weaker anti-RBD IgG level: 302 ± 291 (SD: 965) BAU/mL than 928 ± 215 (SD: 1324) BAU/mL without CD20 blocking in the HD group, ^**^p < 0.01 (Figure 1D). Eleven (73%), or sixteen (100%), or fifteen (83%) HD cases were diagnosed more than 1 year ago of the withdrawal of the blood. There was no statistical difference among the types of vaccines in terms of SARS-CoV-2 reactive IgG production due to disease duration following diagnosis (Figure 1E).

### 3.2. SARS-CoV-2 specific cellular immunity in HDs following vaccination

Next, the SARS-CoV-2 reactive cellular immunity was assayed by flow cytometry. The PBMCs were reactivated with SARS-CoV-2 antigens (S-, M-, N-peptide pools) and CD4^+^ or CD8^+^ T-cells were assayed to produce TNF-α, IFN-γ, and CD40L *ex vivo*. The cellular response rate corresponds to the percentage of subjects in one group in terms of the TNF-α, IFN-γ, or CD40L cytokine producing CD4^+^ or CD8^+^ T-cells over the cut off value that is 400 reactive cells of 10^6^ parental population (Supplementary Figure S2). The simple “response” in Results 3.2 corresponds to the number of S-M-N-peptivator activated T-cells normalized to the background unstimulated controls. There was no statistical difference in the absolute number of reactive CD4^+^ TNF-α T-cells in HDs versus HCs. However, as a trend HDs showed weaker CD4^+^ TNF-α^+^ T-cell activation, responsive cells (mean ± SEM/1 × 10^6^ parental CD4^+^ T-cells) were 814 ± 491 (SD: 1552) for HC versus 153 ± 73 (SD: 293) for HDs in the AZD1222 vaccinated group. The mRNA-based BNTB162b2 vaccination resulted in the activation of 382 ± 148 (SD: 591) reactive CD4^+^ TNF-α T-cells in the HC group vs. 161 ± 55 (SD: 235) cells in HDs (Figure 2A). The number of CD4-TNF-α positive cells was highest in the BBIBP-CorV vaccine group: 2082 ± 1104 (SD: 3982) in HCs and 1955 ± 1655 (SD: 6079) in HDs (Figure 2A). Measurement of IFN-γ induction in CD4^+^ T-cells showed also higher SARS-CoV-2 specific activation in HCs vs. HDs, detecting for BBIBP-CorV: 1767 ± 911 (SD: 3286) vs. 190 ± 57 (SD: 213) (^*^p < 0.05); for AZD1222: 1619 ± 950 (SD: 3005) vs. 110 ± 33 (SD: 131); and for BNT162b2: 191 ± 75 (SD: 301) vs. 103 ± 31 (SD: 130) reactive CD4^+^ IFNγ T-cells, respectively (Figure 2B). Then CD40L expression was significantly higher in CD4^+^ T-cells in the HC vs. HDs for BBIBP-CorV: 2511 ± 849 (SD: 3062) vs. 323 ± 145 (SD: 543) ^***^p < 0.001; and for AZD1222: 1980 ± 912 (SD: 2884) vs. 193 ± 94 (SD: 377) ^**^p < 0.01, respectively (Figure 2C). The BNTB162b2 vaccine caused also higher CD40L activation in CD4^+^ T-cells in HCs vs. HDs, namely 630 ± 224 (SD: 895) vs. 286 ± 123 (SD: 522) reactivated cells were detected, but it was not statistically significant difference (Figure 2C). Interestingly the inactivated virus vaccine BBIBP-CorV induced significantly higher CD40L response in HCs vs. BNT162b2: 2511 ± 849 (SD: 3062) vs. 630 ± 224 (SD: 895) ^*^p < 0.05 (Figure 2C).

**Figure 2:** The CD4^+^ T-cells were assayed following ex vivo S/M/N peptide stimulation. Subjects in HCs vs. HDs were for BBIBP-CorV-2 *n* =13 vs. *n* = 16; for AZD1222 *n* = vs. *n* = 16; for BNT162b2 *n* = 16 vs. *n* = 18. The reactive TNF-α **(A)**, or IFN-γ **(B)**, or CD40L **(C)** producing CD4^+^ T-cells were quantified. Mean (red line) and SEM (green whisker) values are demonstrated. ^*^p < 0.05, ^**^p < 0.01, and ^***^p < 0.001.

The cellular response rate, the proportions of CD4^+^ TNF-α^+^ responder cases to vaccination in HC vs. HD for BBIBP-CorV were 61.5% vs. 35.7%; 40% vs. 12.5% for AZD1222, and 25% vs. 16.7% for BNT162b2. The proportions of CD4^+^ IFN-γ^+^ responders in HC vs. HD for BBIBP-CorV were 61.5% vs. 21.4%; for AZD1222 40% vs. 6.3%; for BNT162b2 18.8% vs. 5.6%. The proportions of CD4^+^ CD40L^+^ responders in HC vs. HD for BBIBP-CorV were 92.3% vs. 21.4%, for AZD1222 80% vs. 18.8%, and for BNT162b2 50% vs. 22.2% (Supplementary Figures S2A–C).

**Figure 3:**
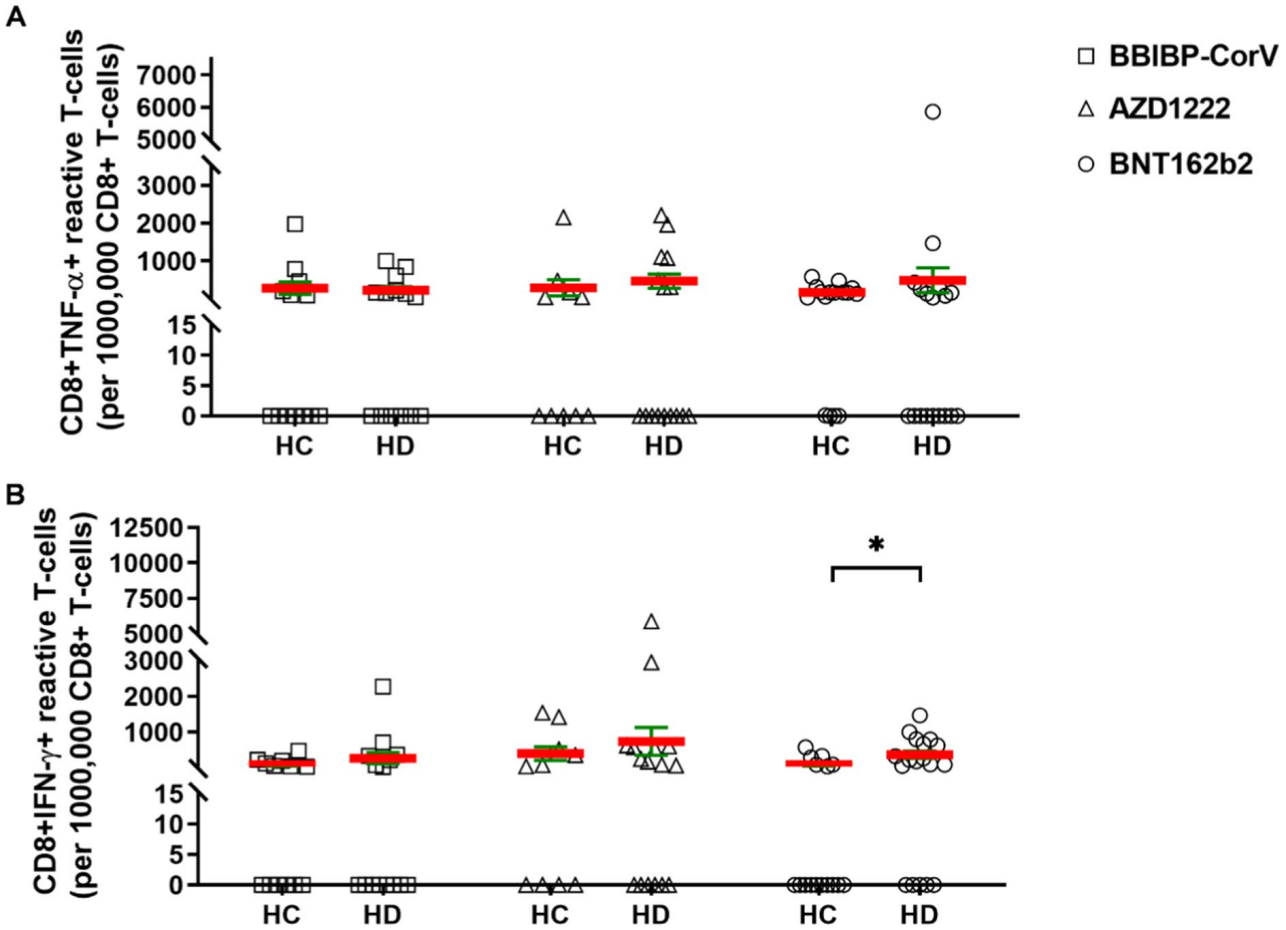
The CD8^+^ T-cell responses in HCs vs. HDs receiving different vaccines. The CD8^+^ T-cells were assayed following *ex vivo* S/M/N peptide stimulation. Subjects in HCs vs. HDs were for BBIBP-CorV-2 *n* = 13 vs. *n* = 16; for AZD1222 *n* = 10 vs. *n* = 16; for BNT162b2 *n* = 16 vs. *n* = 18. The reactive TNF-α **(A)**, or IFN-γ **(B)** producing CD8^+^ T-cells were quantified. Mean (red line) and SEM (green whisker) values are demonstrated. ^*^p < 0.05, ^**^p < 0.01, and ^***^p < 0.001.

The cytotoxic CD8^+^ T-cell mediated cellular immunity was measured for the induction of TNF-α or IFN-γ production. The HDs showed not significantly but tendentiously higher SARS-CoV-2 reactive CD8^+^ TNF-α T-cell reactivation vs. HCs for AZD1222: 466 ± 183 (SD: 734) vs. 288 ± 213 (SD: 674); and for BNT162b2: 485 ± 327 (SD: 1387) vs. 169 ± 43 (SD: 171) (Figure 3A). In BBIBP-CorV vaccine group CD8^+^ TNF-α positive cells were comparable in HCs and HDs: 277 ± 158 (SD: 561) vs. 225 ± 90 (SD: 337) (Figure 3A). The IFN-γ response in CD8^+^ T-cells was also higher in HDs vs. HCs as a tendency for BBIBP-CorV: 265 ± 153 (SD: 593) vs. 86 ± 38 (SD: 139); for AZD1222: 733 ± 389 (SD: 1556) vs. 392 ± 189 (SD: 599); and for BNT162b2: 363 ± 101 (SD: 429) vs. 87 ± 41 (SD: 165) ^*^p < 0.05, respectively (Figure 3B).

The proportions of CD8^+^ TNF-α^+^ responder cases to vaccination in HC vs. HD for BBIBP-CorV were 23.1% vs. 21.4, 20% vs. 31.3% for AZD1222, and 12.5% vs. 16.7% for BNT162b2. The proportions of CD8^+^ IFN-γ^+^ responders in HC vs. HD for BBIBP-CorV were 7.7% vs. 14.3%; for AZD1222 30% vs. 37.5%; for BNT162b2 12.5% vs. 33.3% (Supplementary Figures S2D–E).

**Figure 4:**
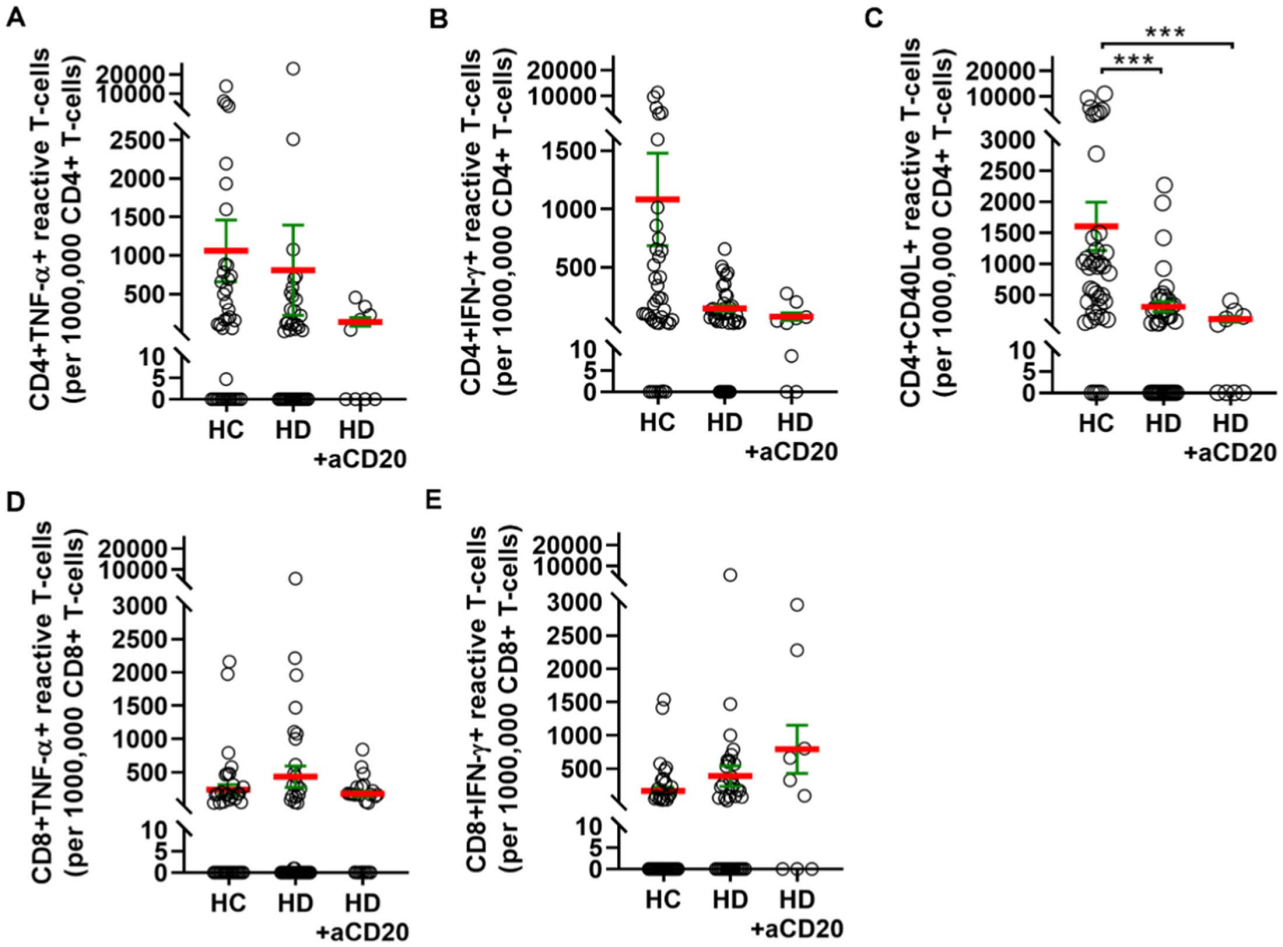
T-cell mediated SARS-CoV-2 specific peripheral immunity in HCs (*n* = 39), HDs (*n* = 39) or HDs that underwent anti-CD20 therapy (*n* = 9). The reactive TNF-α **(A)**, or IFN-γ **(B)**, or CD40L **(C)** producing CD4^+^ T-cells were quantified. The reactive TNF-α **(D)**, or IFN-γ **(E)** producing CD8^+^ T-cells were quantified. Cases vaccinated with BBIBP-CorV, or AZD1222, or BNT162b2 were pooled to investigate the effect of anti-CD20 therapy on T-cell mediated immunity. **(C)** The anti-CD20 therapy significantly reduced the CD4^+^ CD40L^+^ reactive cell numbers in HDs.

Next, we aimed to investigate the effect of anti-CD20 therapy on T-cell mediated peripheral immunity. Pooling cases both in the HC or HD groups irrespective of the vaccination type and dividing HDs based on anti-CD20 therapy status into two groups, the monoclonal antibody treatment in HDs tendentiously resulted in further weaker reactivation of CD4^+^ T-cells upon SARS-CoV-2 antigen exposure. The numbers of CD4^+^ TNF-α^+^ reactive cells were 1060 ± 401 (SD: 2507) in HCs, 807 ± 587 (SD: 3668) in HDs without anti-CD20 therapy, or 134 ± 57 (SD: 170) in HDs with anti-CD20 therapy (Figure 4A). The numbers of CD4^+^ IFN-γ^+^ reactive cells were 1083 ± 397 (SD: 2481) in HCs, 144 ± 27 (SD: 169) in HDs without anti-CD20 therapy, or 74 ± 32 (SD: 97) in HDs with anti-CD20 therapy (Figure 4B). The numbers of CD4^+^ CD40L^+^ reactive cells were 1603 ± 391 (SD: 2443) in HCs, that was significantly reduced to 304 ± 83 (SD: 520) in HDs (^***^p < 0.001) without anti-CD20 therapy, or further decreased in HDs with anti-CD20 therapy 101 ± 47 (SD: 141) (^***^p < 0.001) (Figure 4C). The CD8^+^TNF-α^+^ or CD8^+^ IFN-γ^+^ reactive, SARS-CoV-2 specific T-cell numbers did not differ significantly among the HCs and HDs with no significant effect of anti-CD20 therapy (Figures 4D–E). The virus reactive CD8^+^ IFN-γ cells were slightly increased in the anti-CD20-treated HD group compared to “untreated” HDs and HCs (Figure 4E).

## 4. Discussion

Patients with hematological malignancies represent a specifically susceptible population to COVID-19 with a heightened risk of severe disease and fatality (1–3). As the COVID-19 vaccination campaigns around the world unfolded and studies with large cohorts of HD patients arose, robust data was collected regarding vaccination efficacy and safety in HD patients. However, most trials investigated the effect of mRNA-and vector-based vaccines. Therefore, information on inactivated vaccines is limited. However, inactivated virus vaccines against SARS-CoV-2, such as BBIBP-CorV (Sinopharm, China), CoronaVac (Sinovac, China), Covaxin (India), COVIran Barekat (Iran), contributed significantly to worldwide vaccine coverage (22, 23). In Hungary, BBIBP-CorV was administered at large scales besides other vaccines, even to people at higher risk, like the elderly and patients with various cancers, including hematological malignancies. To the best of our knowledge, only one study addressed the efficacy of the BBIBP-CorV vaccine in cancer patients (24), and a comprehensive analysis of different vaccine types, including BBIBP-CorV in the context of HD is completely lacking. Here, we report our findings about the efficacy of the BBIBP-CorV vaccine compared to mRNA vaccine BNT162b2 (Pfizer BioNTech) and vector-based AZD1222 (AstraZeneca) vaccines to trigger humoral and cellular immunity in patients with hematological disease.

The receptor binding domain (RBD) specific anti-spike (S) IgG isotype antibodies were measured in HDs versus healthy controls following complete vaccination after 4 months. The highest antibody titers were attained in the BNT162b2 group (both controls and HDs), independent of disease status. Due to varying BAU/mL values within each group and the small size of the groups, no significant difference was observed between healthy controls and HDs in the case of any of the examined vaccine types (Figure 1A). The calculated seroconversion rates (Figure 1B) were more informative about the humoral response of HDs upon different vaccines than raw anti-RBD antibody values. Namely, seroconversion rates of HDs fell behind that of matching healthy controls: 33% vs. 69% (BBIBP-CorV), 56% vs. 93% (AZD1222), 72% vs. 100% (BNT162b2). This decreased performance of BNT162b2 and AZD1222 in HD patients is consistent with a pooled humoral response rate of 67.7% of HD patients found in the latest meta-analysis, where data was extracted mainly from articles on mRNA and adenoviral vaccines (9). The proportion of BBIBP-CorV vaccinated HDs mounting anti-S IgG response was only 33% which is comparable with the 38.1% from the only previous report by Ariamanesh and coworkers (24). The observed waning humoral response in HDs is in line with literature data affected by several patient factors such as age, sex, serostatus, treatments and comorbidities (25, 26). It should be noted that 5 out of 15 patients (33.3%) vaccinated with BBIBP-CorV received anti-CD20 therapy. On the other hand, 2 out of 16 (12.5%) and 4 out of 18 (22.22%) patients vaccinated with AZD1222 or BNT162b2 were on anti-CD20 therapy, respectively. Thus, anti-CD20 therapy was overrepresented in the BBIBP-CorV group, which could skew the vaccination response in this group. There is a clear consensus that immunosuppressive B-cell-directed treatments markedly impair immunogenicity in HD patients. Monoclonal anti-CD20 therapy in HD patients was associated with a considerably lower or undetectable humoral response due to prolonged B-cell depletion. Seroconversion of these patients was even poorer if the anti-CD20 was administered within 6–12 months before vaccination (10–14). Therefore, the impaired humoral response of HD patients undergoing anti-CD20 therapy in our cohort was expected, which manifested in significantly lower (^**^p < 0.01) anti-RBD IgG levels in patients treated with anti-CD20 (Figure 1D). This effect was observed with all the studied vaccine types (Figure 1C), although the low number of subjects per vaccine type does not allow statistical comparison and drawing clear conclusions.

It has been emphasized that adding cellular response metrics to the gold standard antibody titer and neutralizing activity measurements would provide a better insight into the overall immune response to COVID-19 vaccination. However, cellular assays are more logistically complex to conduct compared to antibody measurements because they require viable PBMC samples with longer and more complex handling protocols (27). Both ELISpot (enzyme-linked immune absorbent spot) and flow cytometric assay are used to determine the magnitude of cellular response by measuring the ratio of cytokine-secreting cells within the PBMC population after antigen-specific activation. In contrast, flow cytometry has the extra advantage of providing additional information about the type of responding cells. Such studies incorporating flow cytometry assays to compare the cellular immune memory elicited by COVID-19 vaccines are limited, especially those with a simultaneous side-by-side comparison of multiple vaccine types.

Here, we compared SARS-CoV-2 specific T-cell responses induced by BNTB162b, AZD1222, and BBIBP-CorV in HD patients versus healthy individuals by flow cytometry. The strength of the current work is the separate enumeration of responsive CD4^+^ and CD8^+^ T-cells based on IFN-γ/TNF-α/CD40L expression. There was no significant difference in CD4^+^ TNF-α^+^ or CD4^+^ IFN-γ^+^ T-cell frequencies between HCs and HDs except for higher CD4^+^ IFN-γ^+^ T-cells in HCs vs. HDs in BBIBP-CorV group (Figure 2B), although, in the HD group, the % of responders above the cut-off value of SARS-CoV-2 specific T-cells was lower in all vaccine groups. The frequency of CD4^+^ CD40L^+^ T-cells in HD patients was significantly lower compared to healthy volunteers in BBIBP-CorV and AZD1222 vaccine groups. In the case of BNTB162b, the proportion of CD4^+^ CD40L^+^ T-cells was also lower, but the difference was not significant (Figure 2C). The percentage of responders among healthy individuals was also highest in the CD4^+^ CD40L^+^ group in all vaccines, and in HDs, their proportion was reduced in all vaccine groups. The cytotoxic CD8^+^ T-cell mediated immunity was measured by the induction of TNF-α or IFN-γ production. There was no significant difference in the number of reactive CD8^+^ TNF-α^+^ T-cells between HCs and HDs, but interestingly, their number was higher in HDs vs. HCs in AZD1222 and BNTB162b group (Figure 3A). In HDs, tendentiously more responsive CD8^+^ IFN-γ^+^ T-cells were detected than in HCs in BBIBP-CorV and AZD1222 groups, and the difference was significant in the BNTB162b group (Figure 3B).

For comparison, we enlist the articles addressing SARS-CoV-2 specific T-cell response of people with hematological malignancies, which is only a tiny fragment of studies on mRNA or vector-based vaccines demonstrating cellular immunity after complete vaccination in 29%–88% of HD patients. Majority of the studies applied IFN-γ release assay (16, 17, 28, 29); IFN-γ ELISpoT (10, 12, 30–35); and IFN-γ/IL-2 FluoroSpot assay (36–38) or the combination of these (39). One study evaluated T-cell responses by immunosequencing the TCRβ chain (15). Some of these studies enrolled healthy controls as well and showed that the percentage of HD patients with SARS-CoV-2 reactive IFN-γ secreting T-cells is lower compared to that of healthy volunteers (10, 12, 28, 29, 32–35). Admittedly, our results are difficult to directly parallel with bulk anti-SARS-CoV-2 T-cell responses by these reports. The method used by Ehmsen and co-workers enabled the dissemination of CD4^+^ restricted IFN-γ responses from CD4^+^ plus CD8^+^ IFN-γ responses in HD patients vaccinated with mRNA vaccines (16). Among patients with HD, 45% exhibited positive IFN-γ responses by T-cells, 81% of whom were positive for both CD4^+^ and CD8^+^ T-cells, and 18% only elicited a CD8^+^ T-cell response (16). Furthermore, a report by Clemenceau and colleagues combined IFN-γ ELISpoT and flow cytometry for quantifying cellular immunity in AML and MDS patients receiving allogeneic stem cell therapy identifying spike-specific IFN-γ and TNF-α secreting cells within CD4^+^ and CD8^+^ T-cell populations. They found a higher percentage of SARS-CoV-2 specific CD4^+^ TNF-α^+^ T-cells in allo-HSCT receiving patients than in healthy controls. However, after pooling the responses in CD4 and CD8 arms, only 78% of patients achieved cellular immunity compared to 100% of controls (40). Finally, another recent study focused on the analysis of SARS-CoV-2 specific CD8^+^ T-cells in CLL and MDS patients after BNTB162b vaccination using DNA-barcoded peptide-MHC multimers covering the full SARS-CoV-2 Spike-protein they were able to map CD8 T-cell recognition sites and identified 59 antigen epitopes. Surprisingly, they also showed a higher frequency of vaccine-induced antigen-specific CD8^+^ T-cells in the patient group than in healthy donors (41). This observation is consistent with our findings on increased SARS-CoV-2 specific CD8 T-cells in the HD group (Figure 3). Based on literature data, the authors may speculate that induction of T-cell mediated cellular responses by inactivated viral vaccine may rely at least partially, on the cross-presentation of viral antigens to MHC-I not exclusively demonstrated on MHC-II leading to CD8^+^ T-cell activation, that is further boosted by the identified CD4^+^ Th1 (IFN-γ^+^and/or TNF-α^+^) helper T-cells (42, 43).’

The effect of anti-CD20 therapy on cellular immunity of hematological malignancy patients to SARS-CoV-2 is rarely presented in the related articles with divergent conclusions. The frequency of T-cell mediated responses were diminished in anti-CD20 treated chronic lymphocytic leukemia (CLL) patients (14%) compared to patients without the immunosuppressive therapy (29%) (32). In another study, majority of the lymphoma patients (75.3%) achieved cellular responses and their frequency was slightly lower (70%) in patients on anti-CD20 therapy (17). In a third study the rate of T-cell responses was not reduced in HD patients on anti-CD20 monotherapy compared to HD patients without anti-CD20 (33). In our cohort, pooled HD patients on anti-CD20 therapy showed tendentiously lower number of SARS-CoV-2 reactive CD4^+^ T-cells compared to HD patients with no anti-CD20 treatment (Figures 4A–C) and this result was further confirmed by the reduced or zero % of patients on anti-CD20 achieving the cut-off value of 400 responding CD4 T-cells in a million (data not shown). SARS-CoV-2 reactive CD8^+^ IFN-γ T-cell numbers in HDs were slightly increased by anti-CD20 therapy but the difference was not significant (Figure 4E). Interestingly, after complete COVID-19 vaccination S-specific CD8 T-cell response rate was also higher in rheumatic disease patients receiving anti-CD20 therapy (81.8%) than in immunocompetent controls (66.7%) (44).

In summary, the present study is a side-by-side comparison of three different vaccine platforms (BNTB162b, AZD1222, and BBIBP-CorV) generating humoral and cellular immunity in a diverse cohort of participants with hematological diseases and in healthy volunteers. It has to be noted that our access to the control group was at younger age 43 ± 12 (median, y) while HD patients were 63 ± 14 (median, y) without significant difference. The humoral immunity to SARS-CoV-2 based on seropositivity values was impaired in patients with HDs in line with existing data in the literature. In HDs, the observed seroconversion rates imply the following order in vaccine efficacy inducing humoral immunity: BNTB162 ≫ AZD1222 ≫ BBIBP-CorV. Anti-CD20 therapy had a detrimental impact on the humoral responses regardless of vaccine type. On the other hand, the proportions of patients among HDs with sufficient SARS-CoV-2 specific T-cells above the cut-off value painted a different picture: CD4 T-cell responses in patients with hematological diseases were reduced, and CD8 T-cell responses were sustained or even elevated in all vaccine group compared to healthy controls. Bange et al. (45) have shown an increased CD8 T-cell mediated SARS-CoV-2 specific immunity in HDs as a compensatory mechanism to deficient humoral response. Vaccine performance hierarchy in HD patients generating CD4 TNF-α and CD4 IFN-γ responses was: BBIBP-CorV ≫ AZD1222 ≫ BNTB162b. Regarding CD4 CD40L responses in HDs based on frequency rates, the performance of the three vaccines was comparable. Furthermore, CD8 T-cell response rates of HDs were highest in the AZD1222 vaccine group. Although the CD20 is a classical B-cell marker, it has been recently elucidated that it can be a meaningful marker of pro-inflammatory T-cells (46–48). The anti-CD20 treatment significantly reduced the S-, M-, N-reactive CD4^+^ CD40L^+^ T-cell numbers in HDs.

Overall, patients with malignant hematologic disease have higher risk of mortality than the general population in the case of COVID-19 infection, which can be aggravated by other risk factors such as age, comorbidities, and immunosuppressive therapies. Despite of the clinically less severe latest variants of SARS-CoV-2, the best-case scenario in COVID-19 disease management is to prevent it, especially in vulnerable populations like hematology patients. In our study, we have demonstrated the significant weaker response to vaccines in immunologically impaired population such as hematology patients. Based on our results, the protective humoral immune activity of hematologic patients against SARS-CoV-2 can be best supported by mRNA-based vaccination with BNTb162b compared to other vaccines in this study. On the other hand, inactivated virus vaccine seems to be a better choice to elevate T-cell mediated cellular activity against SARS-CoV-2. It has to be noted that because of the already known decline in the humoral immune response over time, there is a high need for booster shots to prevent severe COVID-19 especially in immunocompromised patients (19, 49–51). However, data about the induction of humoral and cellular immunity followed by BBIBP-CorV vaccination with a side-by-side comparison of AZD1222 and BNT162b2 is restricted to few cited papers, limitations of the current research should be mentioned such as the followings. (1) The authors had access to patient-derived blood from heterogenous HDs, (2) our study lacks multi-center execution, (3) and the access to the HC group was with younger age median. (4) Latest Omicron subvariants displayed increased evasion of neutralizing antibodies induced by SARS-CoV-2 vaccination compared to the first Omicron and prior variants (52). Further research is warranted to investigate the effects of different SARS-CoV-2 vaccines on each HDs separately, with clear focus on the booster vaccination protocols also. The sequential combinations of different vaccine types could be the answer for the higher humoral and cellular SARS-CoV-2 protective immunity, but more studies need to be conducted to ensure safety and efficacy.

## Data availability statement

The raw data supporting the conclusions of this article will be made available by the authors, without undue reservation.

## Ethics statement

The studies involving human participants and The enrollment of patients were reviewed and approved by the Human Investigation Review Board of the National Public Health Center under Project Identification Code 47226-7/2019EÜIG. The patients/participants provided their written informed consent to participate in this study.

## Author contributions

SM, LP, and GS conceived and designed the study, participated in data collection and analysis. SM supervised clinical data management and organized and supervised the vaccination protocol. NG, LN, AF, and KF performed experiments measuring SARS-CoV-2 specific T-cell responses. LN performed the measurement of SARS-CoV-2 specific neutralizing anti-RBD specific antibodies. SM and BR collected epidemiological and clinical data and assisted with the identification of SARS-CoV-2 infection and follow-up of patients. ES and GS analyzed the data, prepared the figures and wrote the manuscript. LP was responsible for laboratories. GS verified the data and had access to raw data. SM, LH, PN, AB, LP, and GS revised the manuscript. GS and LP had final responsibility for the decision to submit for publication. All authors contributed to the article and approved the submitted version.

## Funding

This research was funded by the 2020‐1.1.6‐JÖVŐ-2021‐00003 and 142877 FK22, KFI_16-1-2017-0105 grant from the National Research, Development, and Innovation Office (NKFI), Hungary. This work was supported by the ÚNKP‐22‐5‐SZTE‐535 New National Excellence Program for GS, and by the KDP-2021 Program for NG (C1764415) of the Ministry for Innovation and Technology from the source of the National Research, Development and Innovation Fund. This work was supported by the János Bolyai Research Scholarship of the Hungarian Academy of Sciences BO/00582/22/8 (GS). This manuscript was prepared with the professional support of SZTE ÁOK-KKA Hetényi Géza Scholarship_5S726 (AB). This manuscript was prepared with the support of the National Young Talent Scholarship for ES (NTP-NFTÖ-21-B-0164) by the Ministry of Culture and Innovation.

## Conflict of interest

AF, LN, and HL were employed by Avidin Ltd. KF was employed by AstridBio Technologies Ltd. LP is the CEO of Avidin Ltd. and Avicor Ltd. GS was employed by CS-Smartlab Devices Ltd.

The remaining authors declare that the research was conducted in the absence of any commercial or financial relationships that could be construed as a potential conflict of interest.

## Publisher’s note

All claims expressed in this article are solely those of the authors and do not necessarily represent those of their affiliated organizations, or those of the publisher, the editors and the reviewers. Any product that may be evaluated in this article, or claim that may be made by its manufacturer, is not guaranteed or endorsed by the publisher.

## Supplementary material

The Supplementary material for this article can be found online at: https://www.frontiersin.org/articles/10.3389/fmed.2023.1176168/full#supplementary-material
