# Supplementary material for: Comparison of humoral and cellular immune responses in hematologic diseases following completed vaccination protocol with BBIBP-CorV, or AZD1222, or BNT162b2 vaccines against SARS-CoV-2
Source: Front Med (Lausanne). 2023 Jul 17;10:1176168. doi: 10.3389/fmed.2023.1176168 (PMC10389666; doi:10.3389/fmed.2023.1176168)
Supplement: Supplementary file 1 [file Data_Sheet_1.docx]

Supplementary Material

Comparison of humoral and cellular immune response in hematologic disorders following two-does vaccination with BBIBP-CorV, or AZD1222, or BNT162b2 vaccines against SARS-Cov-2

**Enikő Szabó^1,†^, Szabolcs Modok^2,†^, Benedek Rónaszéki^2^, Anna Faragó^3,4^, Nikolett Gémes^1,4^, Lajos I. Nagy^3^, László Hackler Jr.^3^, Katalin Farkas^5^, Patrícia Neuperger^1^, József Á. Balog^1^, Attila Balog^6^, László G. Puskás^1,3^* and Gabor J. Szebeni^1,7,8^***

*** Correspondence:** Laszlo G. Puskas e-mail: laszlo@avidinbiotech.com, Gabor J. Szebeni e-mail: szebeni.gabor@brc.hu

# Supplementary Table 1

| **BBIBP-CorV HD group** | **Gender (26.7 % female)** | **Age (years), mean ± SD: 72 ± 5.2** | **Disease duration (years), mean ± SD: 3.8 ± 3.4** | **Therapy: anti-CD20** | **anti-RBD IgG (BAU/ml), mean ± SD: 339 ± 840** |
| --- | --- | --- | --- | --- | --- |
| Multiple myeloma | Male | 72 | 0.50 | no | 979.69 |
| indolent B-cell lymphoma | Female | 63 | 11.25 | yes | 10.90 |
| Chronic myeloproliferative neoplasm | Female | 71 | 2.25 | no | 24.20 |
| Primary myelofibrosis | Male | 79 | 0.92 | no | 667.30 |
| Mantle Cell lymphoma | Male | 64 | 6.33 | no | 10.90 |
| Chronic lymphocytic leukemia | Male | 68 | 4.75 | yes | 10.90 |
| Multiple myeloma | Male | 82 | 1.25 | no | 10.90 |
| Polycythemia vera | Female | 68 | 4.42 | no | 15.70 |
| Diffuse large B cell lymphoma | Male | 70 | 1.66 | no | 10.90 |
| Multiple myeloma | Female | 74 | 4.66 | no | 82.40 |
| Follicular lymphoma | Male | 77 | 6.92 | no | 46.87 |
| Chronic lymphocytic leukemia | Male | 71 | 9.42 | yes | 10.90 |
| Chronic lymphocytic leukemia | Male | 73 | 0.33 | yes | 10.90 |
| Chronic myeloid leukemia | Male | 68 | 2.83 | no | 3193.70 |
| Diffuse large B cell lymphoma | Male | 74 | 0.25 | yes | 10.90 |

Supplementary Table 1. Demographic and clinical characteristics of the BBIBP-CorV vaccinated HD patients.

**2. Supplementary Table 2**

| **AZD1222 HD group** | **Gender (56.3 % female)** | **Age (years), mean ± SD: 53 ± 17.1** | **Disease duration (years), mean ± SD: 4.7 ± 3.0** | **Therapy: anti-CD20** | **anti-RBD IgG (BAU/ml), mean ± SD: 670 ± 1242** |
| --- | --- | --- | --- | --- | --- |
| Chronic myeloid leukemia | Male | 55 | 8.42 | no | 10.90 |
| Hodgkin's lymphoma | Male | 22 | 1.50 | no | 132.54 |
| Marginal zone lymphoma | Female | 58 | 2.92 | no | 12.86 |
| Chronic lymphocytic leukemia | Female | 44 | 4.42 | no | 328.96 |
| Secondary myelofibrosis | Female | 63 | 11.58 | no | 3115.22 |
| Multiple myeloma/PreB-Cell  Acute Lymphoblastic Leukemia | Female | 54 | 5.00 | no | 47.09 |
| Waldenstrom's macroglobulinaemia | Female | 76 | 4.66 | no | 212.11 |
| Hemolytic uremic syndrome | Male | 26 | 1.66 | no | 310.21 |
| Multiple myeloma | Female | 58 | 1.16 | no | 3211.14 |
| Chronic lymphocytic leukemia | Male | 67 | 4.92 | no | 3165.36 |
| Mantle Cell lymphoma | Male | 57 | 10.33 | yes | 10.90 |
| Mantle Cell lymphoma | Male | 41 | 4.00 | no | 10.90 |
| Acute myeloid leukemia | Female | 23 | 3.58 | no | 113.80 |
| Chronic lymphocytic leukemia | Female | 66 | 5.08 | no | 10.90 |
| Chronic lymphocytic leukemia | Male | 74 | 5.08 | yes | 14.39 |
| Mantle Cell lymphoma | Female | 59 | 1.25 | no | 10.90 |

Supplementary Table 2. Demographic and clinical characteristics of the AZD1222 vaccinated HD patients.

3. **Supplementary Table 3.**

| **BNT162b2 HD group** | **Gender (38.8 % female)** | **Age (years), mean ± SD: 65.1 ± 12** | **Disease duration (years), mean ± SD: 4.0 ± 3.2** | **Therapy: anti-CD20** | **anti-RBD IgG (BAU/ml), mean ± SD: 1264 ± 1479** |
| --- | --- | --- | --- | --- | --- |
| Follicular lymphoma (G3) | Male | 70 | 5.16 | no | 3211.14 |
| Marginal zone lymphoma | Male | 69 | 2.75 | yes | 10.90 |
| Myelodysplastic syndrome | Male | 66 | 3.92 | no | 144.97 |
| Diffuse large B cell lymphoma | Female | 27 | 6.00 | no | 338.34 |
| Follicular lymphoma | Female | 69 | 0.16 | yes | 3211.14 |
| Primary myelofibrosis | Male | 62 | 2.00 | no | 3232.94 |
| Chronic lymphocytic leukemia | Male | 71 | 9.25 | no | 10.90 |
| Polycythemia vera | Male | 66 | 5.75 | no | 3215.50 |
| Acute myeloid leukaemia | Female | 56 | 0.83 | no | 10.90 |
| Primary central nervous system (CNS) lymphoma | Female | 64 | 4.50 | no | 3232.94 |
| Chronic lymphocytic leukemia | Female | 74 | 4.08 | yes | 10.90 |
| Chronic lymphocytic leukemia | Male | 80 | 3.33 | no | 3208.96 |
| Myelodysplastic syndrome | Male | 82 | 2.25 | no | 39.46 |
| Multiple myeloma | Male | 70 | 13.58 | no | 83.06 |
| Multiple myeloma | Male | 65 | 3.33 | no | 188.79 |
| Mantle Cell lymphoma | Female | 68 | 1.58 | yes | 10.90 |
| Aggressive T-cell lymphoma | Female | 64 | 0.92 | no | 940.02 |
| Waldenstrom's macroglobulinaemia | Male | 50 | 3.08 | no | 1652.88 |

Supplementary Table 3. Demographic and clinical characteristics of the BNT162b2 vaccinated HD patients.


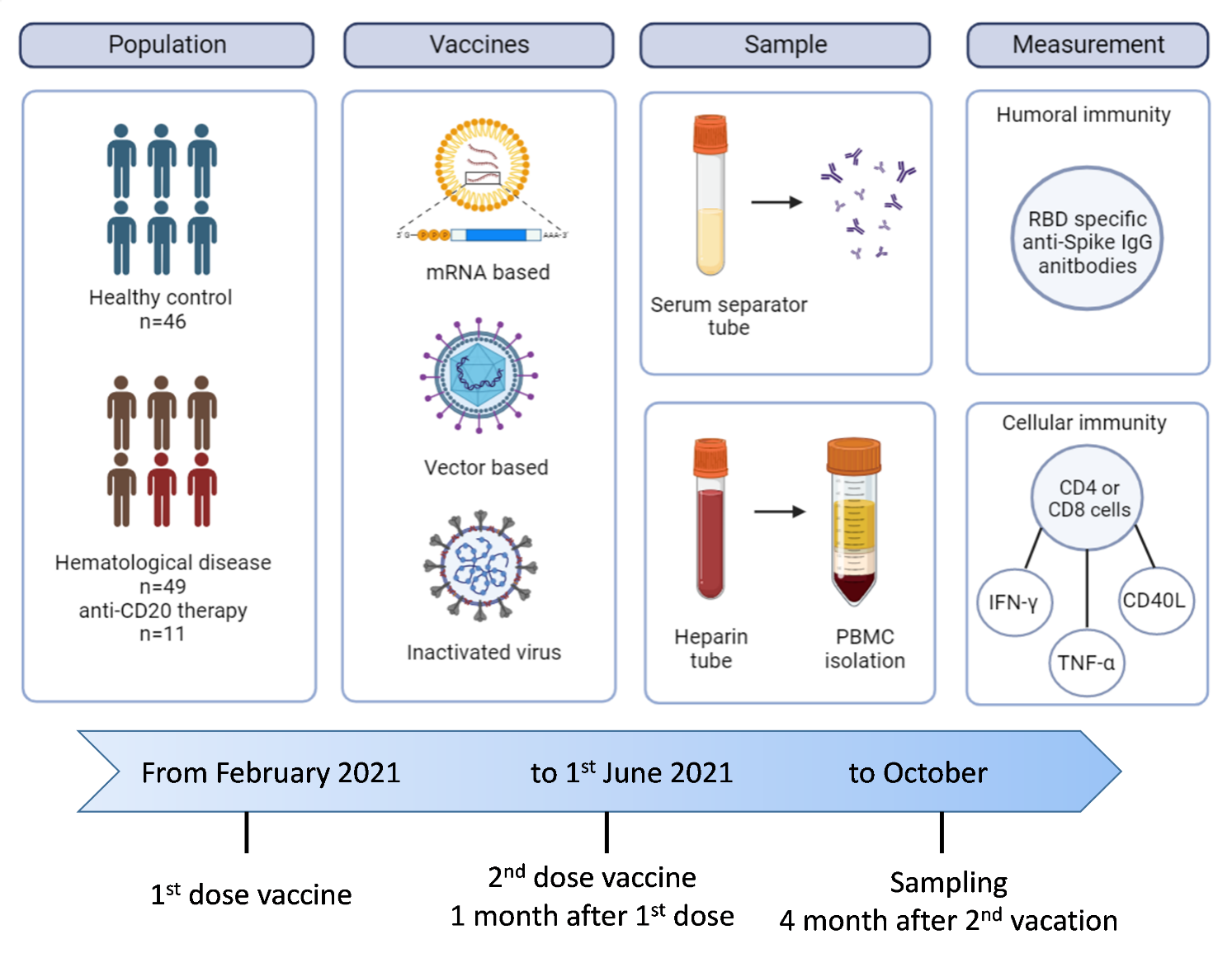


**Supplementary Figure 1.** This prospective observational study was conducted at the Szent-Györgyi Albert Medical School- University of Szeged, Department of Medicine, Szeged, Hungary between October 2021 and February 2021. Adult patients with HDs were recruited who received two doses vaccination starting from February 2021 and completed by 1^st^ June 2021. Peripheral blood and sera sampling was conducted after 4 months of the second vaccination event.


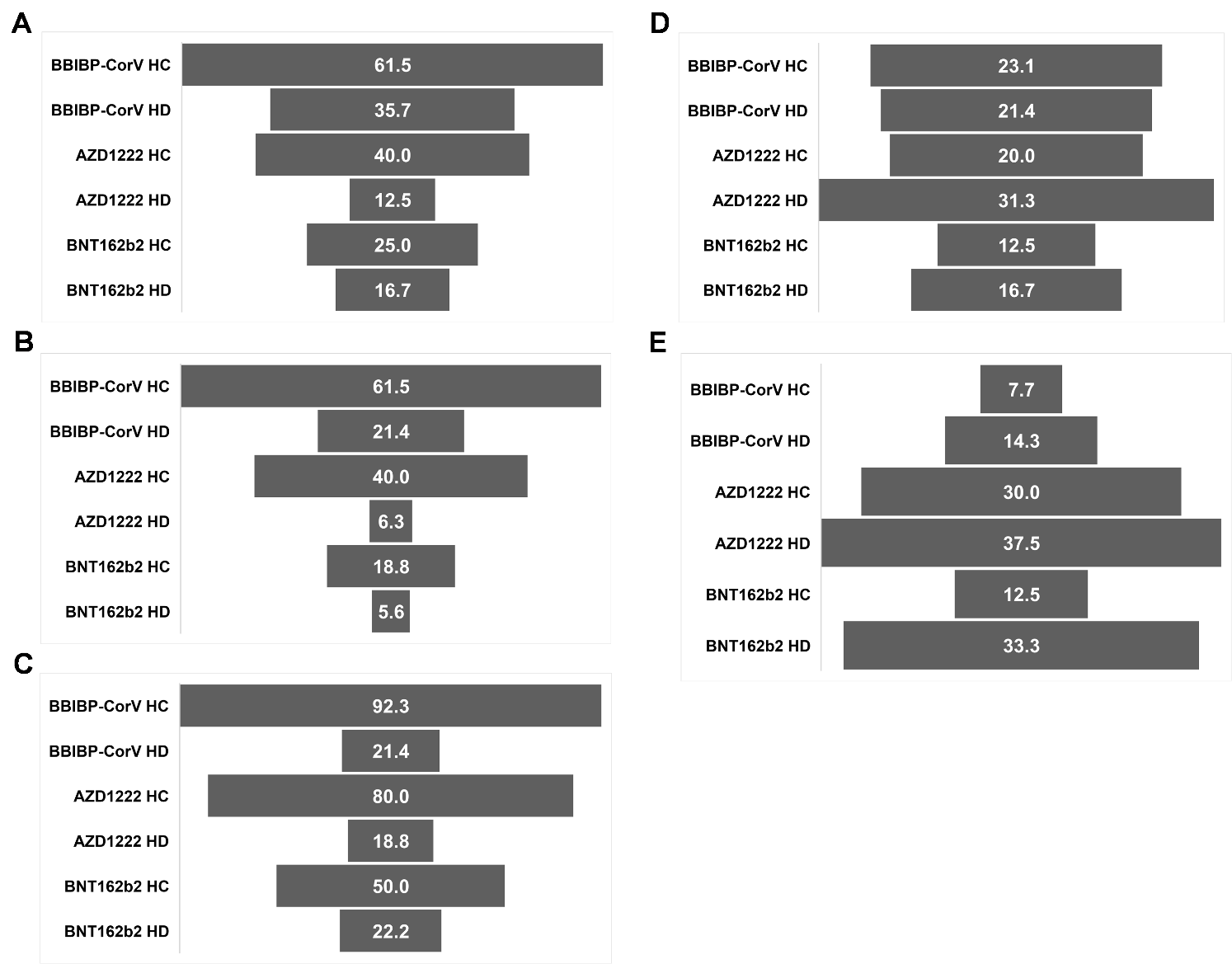


**Supplementary Figure 2.** The response rate of T-cell mediated immunity in HD vs HCs following vaccination with BBIBP-CorV, or AZD1222, or BNT162b2. (A) CD4+ TNF-α+, (B) CD4+ IFN-γ+, (C) CD4+CD40L+, (D) CD8+ TNF-α+, (E) CD8+IFN-γ+ T-cells were assayed by flow cytometry following S-,M-,N- Peptivator stimulation ex vivo. Cell numbers in the reporting gates were normalized to parental CD4+ or CD8+ cells (reactive cell number/parental cell number x 10^6^), then the background was normalized via subtraction of untreated from the stimulated. Finally, reactive cell numbers are shown in relation to 10^6^ CD4+ or CD8+ T-cells (Mean ± SEM/1x10^6^ parental CD4+ T-cells, SD), the cut-off value was 400 reactive cells of 10^6^ parental population. Subjects in HCs vs. HDs were for BBIBP-CorV-2 n=13 vs. n=16; for AZD1222 n=10 vs. n=16; for BNT162b2 n=16 vs n=18.
